# The developmental dopamine trajectory hypothesis of ADHD across the lifespan

**DOI:** 10.3389/fpsyt.2026.1944446

**Published:** 2026-10-07

**Authors:** Raly James Perez Custodio, Stephan Getzmann, Edmund Wascher, Michael C. Pan, Florence Ingred Samante, Fred Lawrence Samante, Bung-Nyun Kim, Hee Jin Kim, Eugene C. Yi, Jae Hoon Cheong

**Affiliations:** 1 Networking Group Aging, Department of Ergonomics, Leibniz Research Centre for Working Environment and Human Factors at TU Dortmund (IfADo), Dortmund, Germany; 2 Experimental Ergonomics, Department of Ergonomics, Leibniz Research Centre for Working Environment and Human Factors at TU Dortmund (IfADo), Dortmund, Germany; 3 Bradford Institute for Health Research, Temple Bank House, Bradford Royal Infirmary, Bradford, United Kingdom; 4 Department of Pharmacy, St. Scholastica’s College Tacloban Inc., Palo, Leyte, Philippines; 5 College of Medicine, University of the Philippines Manila, Manila, Philippines; 6 Division of Child and Adolescent Psychiatry, Department of Psychiatry, College of Medicine, Seoul National University, Seoul, Republic of Korea; 7 Uimyung Research Institute for Neuroscience, Department of Pharmacy, Sahmyook University, Seoul, Republic of Korea; 8 Department of Molecular Medicine and Biopharmaceutical Sciences, Graduate School of Convergence Science and Technology, Seoul National University, Seoul, Republic of Korea; 9 Institute for New Drug Development, College of Pharmacy, Jeonbuk National University, Jeollabuk-do, Republic of Korea

**Keywords:** attention-deficit/hyperactivity disorder, developmental trajectories, dopamine, dopamine transporter, longitudinal studies, neurodevelopment

## Abstract

Attention-deficit/hyperactivity disorder (ADHD) is increasingly recognized as a lifespan neurodevelopmental disorder, yet the mechanisms underlying its changing clinical presentation across development remain poorly understood. Although childhood ADHD is typically characterized by hyperactivity, impulsivity, and inattention, symptom expression frequently evolves during adolescence and adulthood toward greater reward dysregulation, executive dysfunction, motivational impairment, internal restlessness, and changing manifestations of emotional dysregulation. Existing neurobiological models have substantially advanced understanding of ADHD pathophysiology but provide limited explanation for how symptom continuity can coexist with developmental changes in clinical expression. Here, we propose the Developmental Dopamine Trajectory Hypothesis, which posits that DAT related dopaminergic dysregulation may interact with the sequential maturation of frontostriatal, mesolimbic, and mesocortical circuits to contribute to developmental changes in ADHD symptom expression across the lifespan. A central proposition of the framework is that the functional consequences of DAT related dopaminergic dysregulation depend on the developmental state of the neural circuits on which it acts. Within this framework, childhood manifestations may arise primarily from interactions between dopaminergic vulnerability and developing frontostriatal networks, adolescent manifestations from increasing involvement of reward and emotion related circuitry, and adult manifestations from interactions with mature executive control and motivational systems. By integrating evidence from longitudinal clinical studies, neuroimaging, electrophysiology, pharmacology, genetics, and translational DAT models, the proposed framework provides a mechanistic model for how developmental changes in neural circuit state may contribute to changing symptom expression despite continuity of underlying neurobiological vulnerability. The hypothesis generates testable predictions regarding longitudinal symptom trajectories, developmental biomarker changes, potential developmental differences in treatment responsiveness, and adolescence as a proposed critical neurodevelopmental transition period. Reconceptualizing ADHD through a developmental dopaminergic framework places developmental timing and neural circuit maturation at the center of the relationship between dopaminergic vulnerability and clinical phenotype, providing a basis for future longitudinal, mechanistic, and developmentally informed research across the lifespan.

## 1 Introduction: ADHD as a lifespan neurodevelopmental disorder

Attention-deficit/hyperactivity disorder (ADHD) is a prevalent neurodevelopmental condition characterized by persistent inattention, hyperactivity, and impulsivity that impair functioning across multiple domains of life (1). Although historically conceptualized as a childhood disorder, accumulating evidence supports ADHD as a lifespan condition whose manifestations extend well into adolescence and adulthood (1, 2). Affecting approximately 7.2% of children and 2.6% of adults worldwide, ADHD is associated with substantial educational, occupational, social, and psychiatric burden, making it a major public health concern (1). Despite increasing recognition of adult ADHD, research and clinical frameworks have remained largely pediatric in focus, limiting understanding of the mechanisms that underlie developmental continuity and age-related changes in symptom expression (2).

Longitudinal evidence demonstrates that ADHD frequently persists into adulthood, although its developmental course is highly heterogeneous (3). Reported persistence rates range from approximately 39% over 10 years in clinical cohorts to more than 70% when residual symptoms and functional impairment are considered, with recent prospective data indicating diagnostic persistence in 87.5% of affected youth followed into adulthood (2, 4, 5). Importantly, persistence does not imply phenotypic stability. Symptom trajectories often fluctuate, with individuals exhibiting varying patterns of persistence, partial remission, recurrence, and functional adaptation across development (6). While overt hyperactivity commonly declines with age, executive dysfunction, emotional dysregulation, motivational impairments, organizational difficulties, and internal restlessness become increasingly prominent during adolescence and adulthood (2, 7). These developmental shifts suggest that ADHD is better conceptualized as a dynamic disorder characterized by evolving symptom expression rather than a static clinical entity.

Despite substantial advances in ADHD research, its neurobiological basis remains incompletely understood. Multiple lines of evidence implicate neurotransmitter systems, distributed neural networks, genetic influences, and environmental factors, yet findings remain heterogeneous and difficult to integrate into a unified developmental framework (1). Clinical heterogeneity, high rates of psychiatric comorbidity, variable treatment response, and the absence of definitive biomarkers further complicate efforts to establish coherent pathophysiological models (1, 2, 8–10). Although neuroimaging studies have identified structural and functional alterations across multiple brain systems, findings vary according to developmental stage, methodology, and clinical characteristics, and effect sizes are often modest (1, 11). Moreover, prevailing conceptual frameworks of ADHD, including executive dysfunction (12), delay aversion/dual pathway (13), and cognitive–energetic models (14), have largely been informed by cross-sectional observations, providing limited insight into the developmental transitions and compensatory adaptations that may drive age-dependent changes in ADHD presentation (9). Consequently, these frameworks provide only a partial account of the developmental changes in ADHD symptom expression, including the shift from predominantly hyperactive–impulsive behaviors in childhood to the broader executive, motivational, and emotional difficulties that often characterize adult ADHD (15, 16).

Among the candidate neurobiological mechanisms, dopaminergic signaling has emerged as one of the most extensively investigated and theoretically influential systems. Evidence from pharmacological, genetic, neuroimaging, and animal studies consistently implicates dopamine related pathways in ADHD, although simplistic models of global dopamine deficiency are insufficient to account for the disorder’s developmental complexity (17). Instead, converging findings suggest that dopamine regulation interacts dynamically with neural maturation and environmental influences to shape symptom expression across the lifespan (1, 17). Within this broader dopaminergic system, the dopamine transporter (DAT) is of particular interest because it is a principal regulator of extracellular dopamine availability through the reuptake and clearance of dopamine, thereby influencing the magnitude and duration of dopaminergic signaling. This regulatory position provides a plausible mechanistic link through which alterations in DAT function could interact with the maturation of dopamine sensitive neural circuits and produce changing effects across development.

The central proposition of the present framework is therefore not simply that DAT related dopaminergic dysregulation contributes to ADHD, but that its functional and clinical consequences may depend on the developmental state of the neural circuits on which it acts. Building on this premise, we propose the Developmental Dopamine Trajectory Hypothesis, which proposes that persistent DAT dysregulation may interact with age dependent neurodevelopmental processes to contribute to changing clinical manifestations across the lifespan. Rather than viewing ADHD as a disorder defined by fixed neurobiological abnormalities, this framework conceptualizes its developmental course as the consequence of evolving functional effects of DAT dysregulation within maturing dopaminergic and corticostriatal circuits.

### 1.1 The unresolved biology of adult ADHD

The recognition that ADHD frequently persists into adulthood has fundamentally challenged its traditional characterization as a childhood-limited disorder (18, 19). However, developmental continuity does not imply phenotypic stability. Accumulating longitudinal and clinical evidence indicates that adult ADHD differs substantially from its childhood presentation, suggesting that the persistence of ADHD across the lifespan may involve developmental changes in the neural systems underlying symptom expression rather than simple continuation of childhood neurobiological processes (20). One of the most consistent developmental observations is the transformation of symptom profiles. Whereas childhood ADHD is typically characterized by overt hyperactivity and impulsive behavior, these manifestations often become less externally visible with age and instead emerge as subjective restlessness, mental overactivity, difficulty relaxing, and persistent inner tension (21, 22). Emerging evidence further suggests that clinically relevant features such as mind-wandering, time blindness, effortful self-regulation, and internal restlessness remain underrepresented within existing diagnostic frameworks despite their prominence in adults with ADHD (23). Together, these findings indicate that symptom persistence is accompanied by substantial changes in clinical expression rather than simple continuation of childhood manifestations.

This developmental shift is paralleled by the increasing prominence of executive dysfunction and emotional dysregulation as major determinants of adult impairment (24). Executive functions, including planning, organization, working memory, inhibitory control, cognitive flexibility, self-monitoring, and goal-directed behavior, become particularly important as academic, occupational, and social demands increasingly rely on higher-order cognitive control systems (25). In a large clinical sample, nearly half of adults with ADHD exhibited clinically significant executive dysfunction, which was associated with greater symptom severity, increased psychopathology, poorer quality of life, elevated emotional dysregulation, and greater mind-wandering (26). However, executive dysfunction alone does not fully capture the adult phenotype. Emotional dysregulation, including emotional lability, irritability, impulsive emotional reactions, low frustration tolerance, and prolonged recovery from emotionally salient experiences, is highly prevalent and contributes substantially to functional impairment, psychiatric comorbidity, and adverse social and occupational outcomes (27, 28). Consistent with this view, emotional instability has emerged as one of the most prominent symptom dimensions among newly diagnosed adults and is strongly associated with overall impairment (21). Qualitative studies similarly identify emotional lability, sleep disturbances, altered time perception, and reduced activation as clinically meaningful features extending beyond the traditional diagnostic symptom triad (29).

Collectively, these observations expose a major limitation of current mechanistic models of ADHD. Most biological theories were developed to explain childhood manifestations of inattention, hyperactivity, and impulsivity and therefore provide limited insight into why symptom profiles systematically transform across development (25). The absence of definitive biological markers, coupled with substantial heterogeneity in symptom presentation and functional outcomes, further highlights the limitations of existing explanatory frameworks (25). Rather than representing simple persistence of childhood symptoms, adult ADHD may reflect a developmentally transformed phenotype characterized by internalized hyperactivity, executive dysfunction, and emotional dysregulation. A central unresolved question is therefore how persistent neurobiological vulnerability may produce different functional consequences as neural circuits mature and the cognitive, social, and environmental demands placed on those circuits change across development. Addressing this question requires developmentally informed models that identify specific neurobiological mechanisms while accounting for the developmental state of the neural circuits on which those mechanisms act. This perspective provides an important foundation for examining whether DAT related dopaminergic dysregulation may contribute to changing ADHD phenotypes across the lifespan.

### 1.2 Dopamine transporter dysregulation as a developmental mechanism in ADHD

Dopaminergic signaling remains one of the most extensively investigated neurobiological systems in ADHD. Evidence from pharmacological, genetic, neuroimaging, and translational studies consistently implicates dopamine-related pathways in symptom expression and behavioral regulation, although contemporary findings no longer support simplistic models of global dopamine deficiency (30). Instead, emerging frameworks emphasize dysregulated dopamine signaling across neural circuits and developmental stages, suggesting that the behavioral consequences of dopamine alterations depend on the maturation state of the affected systems (30). Within this broader dopaminergic system, the dopamine transporter (DAT) represents a particularly relevant candidate mechanism because it regulates extracellular dopamine availability through presynaptic reuptake and thereby influences the magnitude, duration, and temporal dynamics of dopaminergic signaling (30). This regulatory position provides a plausible mechanistic link through which alterations in DAT function may interact with the maturation of dopamine sensitive neural circuits and contribute to changing effects across development.

Dopaminergic processes do not operate in isolation. Dopamine signaling interacts with noradrenergic, glutamatergic, and GABAergic systems that collectively contribute to attention, executive control, motivation, reward processing, behavioral regulation, and neural circuit maturation (31). These interactions provide an important broader neurochemical context for understanding how DAT related alterations may influence ADHD phenotypes across development.

The distinction between tonic and phasic dopamine signaling may be especially relevant to ADHD pathophysiology. Tonic dopamine provides a relatively stable background level of neurotransmission that modulates neural excitability and motivational state, whereas phasic signaling consists of rapid bursts associated with reward prediction, salience attribution, reinforcement learning, and adaptive behavioral responses (32). Effective cognitive and behavioral regulation depends on the dynamic interaction between these signaling modes. Disruption of this balance has been linked to impairments in reinforcement learning, attentional regulation, executive control, and goal directed behavior and is consistent with evidence that individuals with ADHD exhibit altered reward sensitivity, preference for immediate reinforcement, and difficulty sustaining behavior in response to delayed outcomes (32). These findings suggest that altered DAT function may contribute to ADHD not through a uniform reduction in dopamine signaling, but through disruption of the dynamic regulation of dopamine neurotransmission. Because DAT plays a central role in controlling extracellular dopamine availability, DAT dysregulation may disturb the balance between tonic dopamine signaling and phasic dopamine responses to behaviorally salient stimuli. Such dysregulation may impair reinforcement learning, motivational processing, and executive self-regulation, thereby contributing to the heterogeneous cognitive and behavioral manifestations of ADHD.

Importantly, the consequences of DAT dysregulation must be considered within a developmental framework. Dopaminergic systems undergo substantial maturation throughout childhood, adolescence, and early adulthood, including changes in receptor expression, neurotransmitter regulation, synaptic organization, and corticostriatal connectivity (30). The corticostriatal system is particularly relevant because it mediates attention, inhibitory control, reward processing, motivation, executive functioning, and behavioral regulation, and is consistently implicated in ADHD by genetic, transcriptomic, epigenetic, and neuroimaging evidence (30, 33). Recent postmortem findings further identify epigenetic alterations in genes involved in neurodevelopment, neurogenesis, and cellular differentiation within these circuits (33). Together, these observations indicate that the effects of DAT dysregulation may vary according to the developmental state of dopaminergic and corticostriatal networks rather than remain biologically static.

The central implication is that the functional consequences of DAT-related dopaminergic dysregulation may depend not only on the nature or magnitude of the alteration itself, but also on the developmental state of the neural circuits on which it acts. Accordingly, persistent DAT dysregulation may interact with age dependent changes in dopamine signaling and corticostriatal maturation to contribute to distinct clinical manifestations across the lifespan. The same underlying dopaminergic vulnerability may preferentially contribute to hyperactivity, impulsivity, and inattention during childhood, when regulatory circuits remain immature, but may increasingly manifest as executive dysfunction, motivational dysregulation, emotional disturbances, and internal restlessness as these circuits mature. Such a framework offers a parsimonious explanation for the coexistence of symptom persistence and symptom transformation in ADHD and provides the conceptual basis for the Developmental Dopamine Trajectory Hypothesis advanced herein.

### 1.3 From established ADHD models to genetically defined and humanized DAT models

Animal models have been central to elucidating the behavioral, neurochemical, genetic, and developmental mechanisms underlying ADHD. Given the disorder’s marked heterogeneity, no single model fully recapitulates its clinical complexity; instead, different models capture specific behavioral and neurobiological features, providing complementary insights into pathophysiology (34–36). Importantly, the evolution of ADHD animal research reflects a broader conceptual shift from descriptive behavioral models toward genetically defined models that directly interrogate DAT function. This progression is particularly relevant because it enables investigation of mechanisms that may contribute not only to core ADHD symptoms but also to their developmental transformation across the lifespan. Importantly, these models also provide an opportunity to examine whether the consequences of altered DAT function depend on the developmental state and neural circuit context in which the alteration occurs. **Table 1** summarizes the evidence supporting the Developmental Dopamine Trajectory Hypothesis.

**Table 1:** Major lines of evidence integrated within the developmental dopamine trajectory hypothesis.

Evidence domain	Representative findings	Contribution to the hypothesis
Longitudinal ADHD Studies	High rates of symptom persistence into adulthood Fluctuating developmental trajectories Symptom remission, recurrence, and functional adaptation Phenotypic transformation across development	Supports the premise that ADHD exhibits developmental continuity despite substantial changes in clinical expression across the lifespan.
Adult ADHD Phenotype	Internalized hyperactivity and restlessness Executive dysfunction Emotional dysregulation Motivational impairment	Indicates that adult ADHD may represent a developmentally transformed phenotype rather than a simple continuation of childhood manifestations.
Dopamine and DAT Research	Alterations in DAT related signaling Altered tonic and phasic dopamine signaling Reward processing differences Reinforcement learning abnormalities Clinical responsiveness to stimulant medications	Supports dopaminergic dysregulation, including altered DAT function, as a candidate neurobiological mechanism that may contribute to changing symptom expression across development.
Neurodevelopmental Studies	Continued maturation of dopaminergic systems Frontostriatal circuit maturation during childhood Mesolimbic circuit maturation during adolescence Mesocortical maturation extending into adulthood	Provides the developmental basis for the hypothesis that the functional consequences of DAT related dopaminergic dysregulation may vary according to the maturation state of dopamine sensitive neural circuits.
DAT Animal Models	Spontaneously Hypertensive Rat (SHR): Well-established ADHD-relevant behavioral model with dopaminergic abnormalities DAT knockout (DAT-KO) models DAT heterozygous (DAT-HET) models DAT Val559 humanized models Age, sex, and circuit dependent behavioral and neurobiological effects	Provides mechanistic and translational evidence that alterations in DAT function can produce diverse phenotypes and that their effects may depend on developmental stage, biological context, and neural circuit characteristics.
Broader Neurochemical Systems	Noradrenergic involvement in attention and executive control Glutamatergic involvement in synaptic plasticity and circuit function GABAergic involvement in neural inhibition and circuit maturation Interactions among dopamine and other neurotransmitter systems	Places DAT related dopaminergic mechanisms within a broader neurochemical framework and supports investigation of whether developmental changes in DAT related signaling occur alongside changes in interacting neurotransmitter systems.

Converging evidence from longitudinal clinical studies, adult ADHD research, dopamine biology, developmental neuroscience, and dopamine transporter (DAT) animal models provides the empirical foundation for the proposed framework. The conceptualization of the framework was also informed by the contemporary evaluation of the dopamine hypothesis of ADHD by MacDonald et al. (17), which integrates evidence from human studies and animal models. Collectively, these findings support the proposition that DAT-related dopaminergic dysregulation may interact with the developmental state and maturation of neural circuits to contribute to changing ADHD symptom expression across the lifespan, while potentially maintaining continuity of underlying neurobiological vulnerability. The framework remains a hypothesis and requires empirical testing through longitudinal and mechanistically informed studies.

Among traditional models, the Spontaneously Hypertensive Rat (SHR) remains one of the most extensively validated. SHR animals exhibit hyperactivity, impulsivity, attentional deficits, altered reinforcement sensitivity, increased behavioral variability, and impairments in learning and executive like functions, alongside abnormalities in dopaminergic, noradrenergic, and glutamatergic systems and responsiveness to clinically effective stimulant medications (35, 36). Although these characteristics provide strong face and predictive validity, the SHR phenotype arises from complex polygenic and developmental influences rather than a defined molecular mechanism, limiting its utility for identifying specific processes that may contribute to developmental changes in symptom expression (35). This limitation motivated the development of genetically defined DAT models. Complete DAT deletion in DAT knockout (DAT KO) mice produces profound disturbances in dopamine homeostasis, including markedly elevated extracellular dopamine, altered dopamine synthesis and storage, and compensatory changes in dopamine receptor signaling (37, 38). These neurochemical alterations are accompanied by extreme hyperactivity, impulsivity, impaired inhibitory control, disrupted sensorimotor gating, and cognitive deficits, many of which are attenuated by psychostimulants used in ADHD treatment. While these findings provide strong evidence for a role of DAT in regulating dopamine homeostasis and behavior, complete transporter loss represents a biologically extreme condition unlikely to reflect the degree of DAT alteration observed in most individuals with ADHD (37, 38). DAT heterozygous (DAT HET) models address this limitation by producing partial reductions in transporter availability, demonstrating that moderate alterations in DAT function can affect dopamine homeostasis and that their behavioral and neurochemical consequences may be modified by age and sex (39, 40). Notably, age related adaptations in DAT HET mice suggest that the effects of altered DAT function may change across development rather than remain static (39).

The strongest translational support for a developmental DAT framework comes from the DAT-Val559 knock in mouse, a humanized model based on a functional DAT coding variant originally identified in individuals with ADHD and later associated with other neuropsychiatric conditions (41, 42). Unlike DAT-KO models, DAT-Val559 preserves transporter expression and dopamine uptake while producing anomalous dopamine efflux, resulting in persistent alterations in extracellular dopamine regulation despite an intact transporter (41, 42). Importantly, this abnormal dopamine efflux remains sensitive to methylphenidate and amphetamine, linking the molecular effects of the variant to clinically relevant therapeutic mechanisms (41). DAT-Val559 mice exhibit abnormalities in reward processing, motivation, impulsivity, behavioral flexibility, psychostimulant responsiveness, and corticostriatal signaling, with several effects varying according to brain region and sex (43–45). Together, these findings indicate that the consequences of altered DAT function may be shaped by neural circuitry, biological context, and developmental state, rather than reflecting a uniform dopaminergic abnormality.

Collectively, the progression from SHR to DAT-KO, DAT-HET, and DAT-Val559 models reveals an increasingly refined understanding of ADHD pathophysiology. Whereas traditional models established the importance of dopaminergic mechanisms, genetically defined and humanized DAT models demonstrate that alterations in DAT function can produce diverse behavioral and neurobiological outcomes that vary according to age, sex, genetic background, and neural circuit context. These observations support the possibility that DAT related dopaminergic alterations may have developmental consequences that evolve according to the maturation state of the neural systems in which they operate, rather than producing uniform effects across the lifespan. Accordingly, the DAT-Val559 model provides a particularly informative translational framework for investigating how altered DAT function may interact with ongoing maturation of corticostriatal circuitry, forming an important mechanistic foundation for the Developmental Dopamine Trajectory Hypothesis proposed herein.

### 1.4 The developmental dopamine trajectory hypothesis in ADHD

We propose the Developmental Dopamine Trajectory Hypothesis, which posits that the changing clinical presentation of ADHD across the lifespan may reflect developmental changes in the functional consequences of DAT related dopaminergic dysregulation. Rather than conceptualizing ADHD as a static disorder characterized by a uniform dopaminergic abnormality, this framework views ADHD as a dynamic neurodevelopmental condition in which alterations in DAT mediated dopamine signaling may interact with the sequential maturation of frontostriatal, mesolimbic, and mesocortical circuits to contribute to age dependent clinical phenotypes (17, 46). The central proposition is that the effects of DAT related dopaminergic dysregulation depend not only on the presence or magnitude of the alteration, but also on the developmental state of the neural circuits on which it acts. Thus, developmental timing represents a key explanatory variable linking dopaminergic vulnerability to changing clinical expression across the lifespan.

Importantly, this hypothesis does not propose that ADHD consists of distinct age specific disorders or entirely different symptom profiles across development. Instead, it proposes that persistent DAT dysregulation may represent a shared neurobiological vulnerability, while the clinical expression of core ADHD symptoms evolves as dopamine sensitive neural circuits mature and undergo developmental changes. Consequently, symptom persistence and symptom transformation are conceptualized as complementary outcomes of the same neurodevelopmental process. Core features such as inattention, hyperactivity, and impulsivity remain continuous but may be expressed differently across developmental stages, while additional cognitive, motivational, and emotional manifestations may emerge as increasingly specialized neural systems mature. This developmental perspective distinguishes the proposed framework from models that primarily attribute ADHD to a relatively fixed dopaminergic abnormality by emphasizing the interaction between DAT related signaling and the changing state of neural circuitry across development. **Figure 1** illustrates the proposed Developmental Dopamine Trajectory Hypothesis across the lifespan.

**Figure 1:**
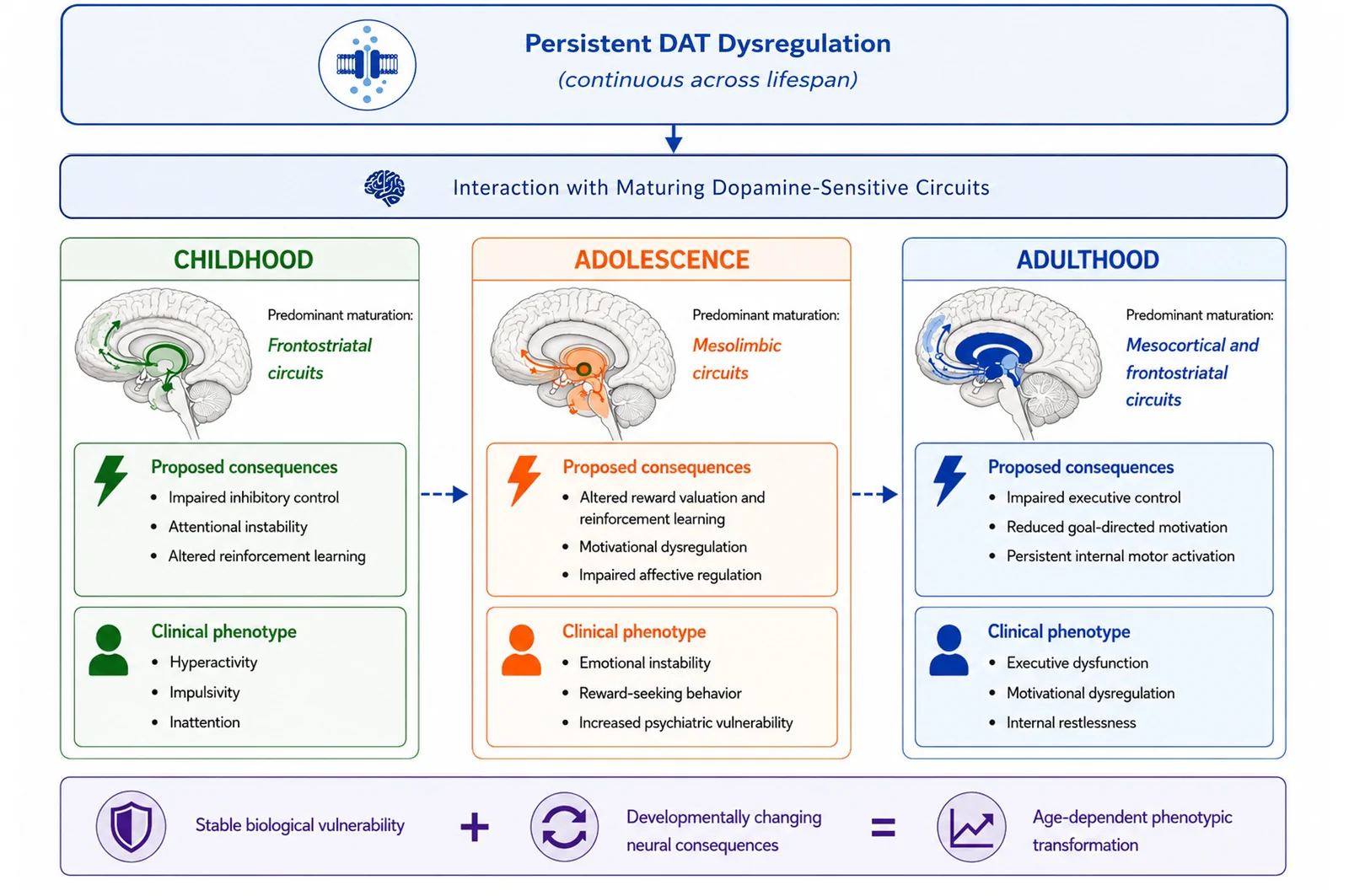
The developmental dopamine trajectory hypothesis of ADHD across the lifespan. Persistent dopamine transporter (DAT) dysregulation is proposed to interact with the sequential maturation of frontostriatal, mesolimbic, and mesocortical circuits, contributing to developmental changes in the clinical expression of ADHD. The framework proposes that the functional consequences of DAT-related dopaminergic dysregulation depend on the developmental state of the neural circuits on which it acts. Thus, a shared neurobiological vulnerability may persist across development, while its functional consequences evolve as dopamine-sensitive neural systems mature. Core ADHD symptoms may persist across development but may be expressed differently across developmental stages, progressing from predominantly overt hyperactivity, impulsivity, and attentional instability in childhood to increasing reward and emotional dysregulation during adolescence and greater executive dysfunction, motivational impairment, and internal restlessness in adulthood. The conceptual framework was informed by the literature on the dopamine hypothesis of ADHD (17). Brain illustrations adapted from Servier Medical Art and SciDraw under the Creative Commons Attribution 4.0 International (CC BY 4.0) license and further modified and annotated by the authors using Microsoft PowerPoint and Inkscape.

#### 1.4.1 Childhood phase

During childhood, the core symptoms of ADHD, including inattention, hyperactivity, and impulsivity, are typically expressed through overt behavioral dysregulation, coinciding with rapid maturation of frontostriatal circuits involved in inhibitory control, motor regulation, and attentional processing (17, 47–49). Within the proposed framework, DAT dysregulation may alter extracellular dopamine regulation within these developing networks, affecting the dynamic balance between tonic dopamine signaling and phasic dopamine responses involved in reinforcement learning, salience attribution, behavioral inhibition, and attentional allocation (17). Consequently, altered dopaminergic modulation may contribute to excessive motor activity, impaired inhibitory control, impulsive behavior, and instability of sustained attention. Because dopamine signaling also contributes to activity dependent synaptic plasticity and neural circuit refinement, altered dopaminergic signaling during this developmental period may influence subsequent circuit maturation and thereby contribute to later changes in symptom expression (17). Thus, the proposed framework considers childhood symptoms not simply as direct consequences of DAT dysregulation, but as manifestations shaped by the interaction between DAT related signaling and the developmental state of frontostriatal circuits.

#### 1.4.2 Adolescent phase

As development progresses into adolescence, the core symptoms of ADHD generally persist, although their behavioral expression often changes. Overt hyperactivity frequently becomes less apparent, whereas impulsivity, attentional difficulties, disturbances in reward processing, emotional dysregulation, and motivational impairment become increasingly prominent (47). This transition coincides with substantial maturation of mesolimbic dopamine pathways connecting the ventral tegmental area, nucleus accumbens, amygdala, and prefrontal cortex, circuits central to reward valuation, motivation, reinforcement learning, and affective regulation (50, 51). Within the proposed framework, the changing maturation state of these networks may modify the functional consequences of DAT dysregulation, potentially contributing to altered reward prediction, delay discounting, reinforcement learning, and motivational processing. Neuroimaging studies demonstrating abnormalities in DAT and D2/D3 receptor availability within reward related regions provide evidence for a role of dopamine dysregulation in these processes (50, 51). Simultaneously, alterations within striato amygdalo medial prefrontal networks may contribute to irritability, affective lability, frustration intolerance, and emotional impulsivity (52–54). These mechanisms may also contribute to the increased vulnerability to anxiety disorders, depressive disorders, conduct problems, and substance use disorders observed during adolescence, with emotional dysregulation representing an important contributor to later psychosocial and psychiatric outcomes (49, 55). Adolescence therefore represents a particularly informative developmental stage for testing whether changes in circuit maturation alter the functional expression of DAT related dopaminergic vulnerability.

#### 1.4.3 Adult phase

In adulthood, ADHD commonly persists despite reductions in overt motor hyperactivity, with the core symptoms increasingly expressed through executive dysfunction, motivational dysregulation, internal restlessness, and persistent deficits in self-regulation (47). According to the Developmental Dopamine Trajectory Hypothesis, these manifestations may reflect the functional consequences of DAT dysregulation within mature mesocortical and frontostriatal networks responsible for higher order cognitive control (48). Consequently, adults with ADHD commonly exhibit impairments in working memory, planning, cognitive flexibility, organization, sustained attention, inhibitory control, and goal directed behavior (48). Alterations within mesolimbic reward circuitry may further contribute to motivational dysfunction. Consistent with this interpretation, positron emission tomography studies have demonstrated altered DAT and D2/D3 receptor availability in reward related regions of adults with ADHD, with these abnormalities correlating with lower achievement motivation and greater attentional impairment (50, 51). Within this framework, internal restlessness represents a developmental expression of persistent hyperactivity rather than its disappearance, manifesting as subjective experiences of mental overactivity, tension, impatience, and difficulty relaxing (2, 47). Likewise, emotional dysregulation may persist across the lifespan and continue to contribute substantially to occupational, social, and interpersonal impairment (52). The adult phenotype therefore illustrates the central proposition of the framework, that the clinical consequences of DAT related dopaminergic dysregulation may change as the neural systems supporting higher order cognitive, motivational, and self-regulatory functions mature.

Collectively, the Developmental Dopamine Trajectory Hypothesis proposes that persistent DAT dysregulation may interact with the sequential maturation of dopamine sensitive neural circuits to contribute to both continuity of neurobiological vulnerability and developmental changes in its clinical expression. Rather than representing distinct age specific disorders, childhood, adolescence, and adulthood represent successive developmental stages in which a shared dopaminergic vulnerability may be expressed through evolving cognitive, behavioral, motivational, and emotional phenotypes. The framework therefore places developmental timing and neural circuit maturation at the center of the proposed mechanism: the consequences of DAT related dopaminergic dysregulation are expected to depend on the developmental state of the circuits on which it acts. **Figure 1** summarizes the proposed interactions among persistent DAT dysregulation, neural circuit maturation, and the developmental evolution of ADHD symptom expression across the lifespan.

## 2 Discussion

### 2.1 Implications for ADHD pathophysiology

The Developmental Dopamine Trajectory Hypothesis reframes ADHD as a dynamic neurodevelopmental disorder in which persistent dopaminergic vulnerability may interact with the maturation of dopamine sensitive neural circuits to contribute to developmental changes in symptom expression across the lifespan (6, 56, 57). Rather than conceptualizing ADHD as a static condition that either persists or remits, the model proposes that clinical manifestations may evolve because the functional consequences of DAT dysregulation vary with developmental stage, the maturation state of neural circuits, environmental demands, and ongoing neurodevelopmental processes (57, 58). This interpretation is consistent with longitudinal evidence indicating that many individuals with ADHD follow fluctuating developmental trajectories characterized by recurrent transitions between symptomatic and remitted states rather than stable persistence or permanent recovery (6, 57).

A central implication of this framework is the distinction between symptom persistence and developmental changes in symptom expression. Traditional approaches have largely defined continuity according to the persistence of diagnostic criteria over time (59). In contrast, the present hypothesis proposes that developmental continuity may reflect a shared neurobiological vulnerability whose functional consequences evolve as dopamine sensitive neural circuits mature. Thus, developmental timing is proposed as a key explanatory variable linking persistent neurobiological vulnerability to changing clinical expression. Under this framework, core ADHD symptoms remain continuous across development but may be expressed differently with age. Emotional dysregulation may likewise persist across the lifespan, although its clinical manifestations may change with developmental stage as neural circuits mature and cognitive, social, and environmental demands increase (24, 28, 60, 61). Thus, predominantly overt hyperactivity, impulsivity, and attentional instability in childhood may be accompanied by increasingly prominent reward dysregulation during adolescence and greater executive dysfunction, motivational impairment, and internal restlessness in adulthood as mesolimbic and mesocortical systems mature (56). This framework provides a potential explanation for why clinically significant impairment may persist despite reductions in overt hyperactivity and why estimates of ADHD persistence vary substantially across diagnostic approaches (5, 59).

The hypothesis also offers a developmental reinterpretation of dopamine dysfunction. Contemporary evidence increasingly argues against a simple dopamine deficiency model and instead supports more complex alterations involving DAT regulation, receptor function, reinforcement learning, and phasic signaling dynamics (17, 62). Because dopaminergic systems continue to mature throughout adolescence and early adulthood, the consequences of persistent DAT dysregulation may vary across developmental stages (63). Consequently, ADHD heterogeneity may arise from variation in the timing, magnitude, and neural localization of developmental dopaminergic alterations interacting with genetic susceptibility and environmental influences (17, 64). Importantly, the framework does not propose that DAT dysregulation alone determines the ADHD phenotype. Rather, it proposes that the developmental state of the neural circuits on which DAT related dopaminergic alterations act may determine their functional and clinical consequences. These dopaminergic processes are also unlikely to operate independently, as interactions with noradrenergic, glutamatergic, and GABAergic systems may further shape attention, executive control, motivation, reward processing, and neural circuit maturation. Collectively, these observations support reconceptualizing ADHD as a disorder characterized by developmentally changing dopaminergic trajectories and their interaction with maturing neural circuits, rather than as a fixed neurochemical deficit. This perspective provides a unified framework for integrating symptom continuity, developmental changes in clinical expression, and phenotypic heterogeneity within a broader neurodevelopmental model (64, 65).

### 2.2 Pharmacological implications across the ADHD lifespan

The Developmental Dopamine Trajectory Hypothesis predicts that pharmacological responses may vary across development because ADHD medications act on neural systems undergoing age dependent maturation. Rather than producing uniform effects across the lifespan, pharmacological interventions may interact with the developmental state of frontostriatal, mesolimbic, and mesocortical circuits, potentially contributing to differences in therapeutic response. Stimulant medications, including methylphenidate and amphetamine-based medications, increase catecholaminergic signaling through effects on dopamine and norepinephrine transport and availability (17, 66). Methylphenidate primarily inhibits dopamine and norepinephrine reuptake, whereas amphetamine-based medications additionally promote catecholamine release and alter transporter mediated neurotransmission. These mechanisms increase extracellular catecholamine availability and can improve attentional control, inhibitory regulation, motivation, and behavioral regulation (67, 68). Stimulants demonstrate established clinical efficacy across childhood, adolescence, and adulthood, although individual treatment response varies according to clinical and developmental factors (67–69).

Within the proposed framework, childhood represents a developmental period in which frontostriatal networks supporting inhibitory control, attentional regulation, and behavioral regulation remain under substantial maturation. Modulation of DAT mediated dopamine signaling by stimulant medications may therefore interact with the developmental state of these networks. During adolescence, continued maturation of mesolimbic and mesocortical circuits coincides with changes in reward processing, emotional regulation, motivation, and psychiatric vulnerability (56, 63). The developmental state of these circuits may therefore influence how pharmacological modulation of catecholaminergic signaling is functionally expressed, potentially contributing to variability in treatment responsiveness, symptom expression, and medication adherence. Persistent intra individual cognitive variability despite stimulant treatment further indicates that some aspects of ADHD related cognitive dysfunction may not be fully normalized by current pharmacotherapies (70).

Importantly, ADHD pharmacology also demonstrates that clinically meaningful treatment effects can occur through mechanisms extending beyond direct DAT modulation. Atomoxetine, a selective norepinephrine transporter inhibitor, primarily enhances noradrenergic signaling and may improve inattention, executive functioning, and impulse control through effects on prefrontal cortical networks (71). Guanfacine and clonidine, which act as α2 adrenergic receptor agonists, provide additional noradrenergic mechanisms that can influence attentional regulation, impulsivity, hyperactivity, and behavioral control (72). The efficacy of these non-stimulant medications indicates that ADHD phenotypes cannot be understood through dopamine signaling alone and that noradrenergic mechanisms contribute to clinically relevant symptom dimensions.

These pharmacological differences provide an important broader context for the Developmental Dopamine Trajectory Hypothesis. Stimulants modify catecholamine availability through DAT and norepinephrine transporter related mechanisms, whereas atomoxetine primarily targets norepinephrine transport and guanfacine and clonidine modulate α2 adrenergic signaling (73). Their clinical effects suggest that dopaminergic and noradrenergic mechanisms may interact within prefrontal and corticostriatal networks. Accordingly, the proposed framework does not imply that DAT dysregulation is the sole determinant of treatment response. Rather, DAT related dopamine regulation is proposed as one component of a broader neurobiological network in which dopaminergic, noradrenergic, glutamatergic, and GABAergic processes interact with the developmental state of neural circuits.

In adulthood, the model predicts continued pharmacological heterogeneity as developmental and clinical factors contribute to diverse phenotypes characterized by executive dysfunction, motivational dysregulation, emotional instability, and internal restlessness rather than overt hyperactivity (56). Variability in treatment outcomes, medication selection, adherence, and response durability is particularly evident in the presence of psychiatric comorbidity (74–76). Methylphenidate and amphetamine-based medications remain important pharmacological options in adults, while atomoxetine and α2 adrenergic agonists provide alternative treatment approaches in appropriate clinical circumstances. These differences in pharmacological mechanism provide a clinically relevant context for examining whether developmental stage and symptom phenotype influence treatment response (67).

Collectively, these pharmacological observations support a developmentally informed and neurochemically pluralistic interpretation of ADHD treatment. The established efficacy of methylphenidate and amphetamine-based medications supports the relevance of catecholaminergic signaling, including DAT mediated dopamine regulation, whereas the efficacy of atomoxetine, guanfacine, and clonidine demonstrates that noradrenergic mechanisms and broader prefrontal and corticostriatal processes also contribute to symptom control. Future longitudinal pharmacological studies should determine whether developmental changes in DAT function, catecholaminergic signaling, and interacting neurotransmitter systems correspond to differences in treatment response across childhood, adolescence, and adulthood. Such studies would directly test whether developmental timing and neural circuit maturation modify the functional consequences of DAT related and other catecholaminergic mechanisms, providing an empirical test of the Developmental Dopamine Trajectory Hypothesis.

### 2.3 Testable predictions

A major strength of the Developmental Dopamine Trajectory Hypothesis is that it generates specific and falsifiable predictions across behavioral, neurobiological, and pharmacological domains. At the behavioral level, the model predicts that ADHD symptoms should undergo systematic developmental transformation rather than simple attenuation over time. Specifically, childhood hyperactivity and impulsivity are expected to evolve into adolescent disturbances in reward processing and emotional regulation and subsequently into adult executive dysfunction, motivational impairment, and internal restlessness (56, 77). Accordingly, longitudinal studies should identify heterogeneous developmental trajectories characterized by symptom transformation rather than a binary distinction between persistence and remission (78, 79).

A particularly important prediction concerns adolescence, which the model identifies as a sensitive neurodevelopmental transition period characterized by substantial changes in neural circuitry, dopaminergic signaling, and developmental trajectories (80, 81). Within this framework, adolescence is proposed as a period during which the interaction between persistent DAT dysregulation and ongoing neural circuit maturation may contribute to pronounced changes in ADHD symptom expression. The key prediction is therefore that the functional consequences of DAT related dopaminergic dysregulation will vary according to the developmental state of the neural circuits on which it acts. Accordingly, longitudinal studies should examine whether age dependent changes in clinical manifestations are accompanied by corresponding trajectories in neurobiological markers associated with dopaminergic function (82). If the proposed framework is correct, these changes would be expected to coincide with the maturation of mesolimbic and mesocortical circuits and may be reflected in longitudinal measures of DAT function, dopamine receptor availability, functional connectivity, or other biomarkers relevant to dopaminergic signaling. Because dopaminergic signaling interacts with noradrenergic, glutamatergic, and GABAergic systems, future studies should also examine whether changes in these systems contribute to developmental variation in ADHD phenotypes (68, 83, 84).

The hypothesis further predicts that developmental stage may influence treatment responsiveness because the neural circuits underlying symptom expression change across the lifespan (85, 86). Consequently, longitudinal studies may identify age dependent differences in the magnitude or pattern of therapeutic response, although the specific mechanisms underlying such differences remain to be established. Such studies should include both stimulant and non-stimulant treatments to determine whether developmental differences in response vary according to pharmacological mechanism, including DAT and catecholaminergic modulation, norepinephrine transporter inhibition, and α2 adrenergic receptor agonism.

Importantly, the Developmental Dopamine Trajectory Hypothesis is directly falsifiable. Longitudinal evidence demonstrates stable symptom expression, invariant biomarker trajectories, and no meaningful developmental differences in treatment responsiveness would challenge the proposed interaction between persistent DAT dysregulation and sequential neural circuit maturation. Likewise, failure to identify adolescence as a period of developmental divergence in clinical manifestations or neurobiological trajectories would weaken one of the model’s central predictions. Conversely, evidence demonstrating developmental changes in symptom expression, age dependent biomarker trajectories, and adolescence as a critical neurodevelopmental transition period would provide empirical support for the proposed framework. Evidence demonstrating that developmental changes in clinical expression correspond to changes in DAT related measures and the maturation state of relevant neural circuits would provide stronger support for the proposed DAT mechanism. In contrast, similar developmental changes occurring independently of DAT related measures would require refinement of the proposed mechanism. **Figure 2** summarizes the principal testable predictions and falsification framework of the Developmental Dopamine Trajectory Hypothesis.

**Figure 2:**
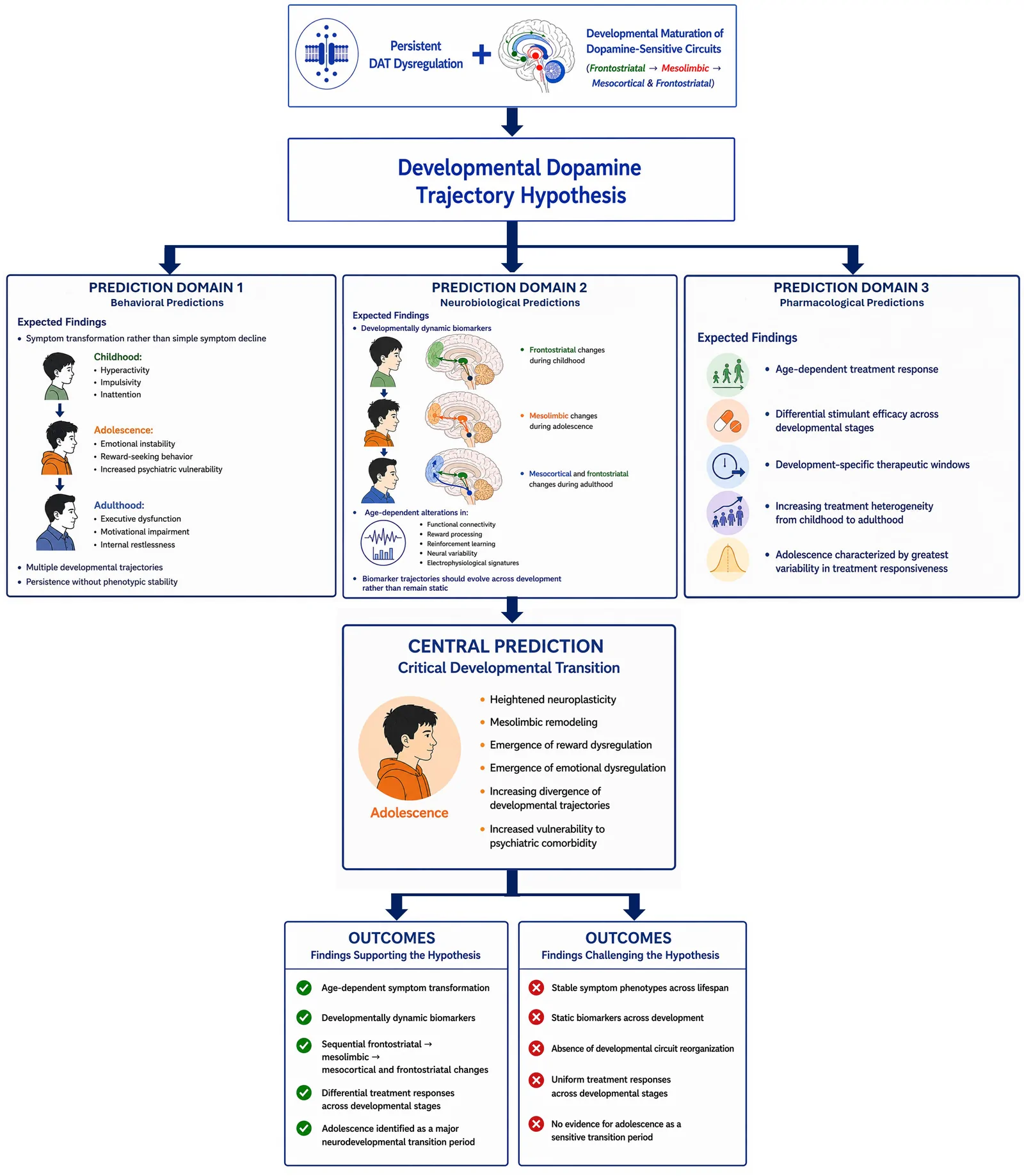
Testable predictions and falsification framework of the developmental dopamine trajectory hypothesis. The model predicts developmental changes in ADHD symptom expression, age-dependent biomarker trajectories, and differential treatment responsiveness across the lifespan arising from interactions between DAT-related dopaminergic dysregulation and neural circuit maturation. The central prediction is that the functional consequences of DAT-related dysregulation vary according to developmental stage and the maturation state of relevant neural circuits. Adolescence is proposed as a critical neurodevelopmental transition period characterized by substantial changes in neural circuitry and increasing divergence in developmental trajectories. Longitudinal evidence demonstrating coordinated changes in symptom expression, DAT-related measures, and neural circuit maturation would provide stronger support for the framework, whereas stable phenotypes, invariant biomarkers, or uniform treatment effects across developmental stages would challenge its central assumptions. Images and illustrations adapted from Servier Medical Art and SciDraw under the Creative Commons Attribution 4.0 International (CC BY 4.0) license and further modified and annotated by the authors using Microsoft PowerPoint and Inkscape.

### 2.4 Future directions

Testing the Developmental Dopamine Trajectory Hypothesis will require longitudinal and mechanistically informed approaches capable of directly tracking developmental changes in dopaminergic function across the lifespan. In animal models, repeated behavioral, neurochemical, molecular, and neuroimaging assessments could determine whether persistent DAT dysregulation is associated with age dependent adaptations in dopamine, noradrenaline, glutamate, GABA, and related signaling systems, helping to clarify how these interacting neurochemical processes contribute to developmental changes in ADHD phenotypes (34, 87, 88). Parallel longitudinal studies in humans incorporating repeated neuroimaging, electrophysiological measures, and cognitive phenotyping will be essential for establishing whether developmental transitions in ADHD symptoms correspond to measurable changes in neural circuitry and neurophysiology. Longitudinal PET or SPECT measures of DAT availability may provide particularly direct tests of the proposed framework by determining whether developmental changes in DAT availability correspond to changes in ADHD symptom expression and the maturation of relevant neural circuits (89). Such designs are particularly important for distinguishing symptom persistence from symptom transformation and for determining whether DAT related changes interact with broader neurochemical and developmental processes, a central prediction of the proposed framework.

A complementary priority is the identification of developmental biomarkers capable of tracking evolving ADHD phenotypes. Emerging evidence suggests that neural variability, functional connectivity, and oscillatory dynamics may provide sensitive indicators of developmental progression in ADHD (90–92). At the molecular level, proteomic studies increasingly implicate pathways related to neurotransmission, inflammation, synaptic plasticity, mitochondrial function, and neurodevelopment, offering a potential bridge between genetic risk and clinical phenotype (93–96). Integrating genomics, transcriptomics, proteomics, neuroimaging, and behavioral measures within longitudinal designs may therefore facilitate identification of biomarkers associated with symptom persistence, remission, and transformation. Importantly, biomarker studies should examine whether developmental changes in these measures covary with both DAT related measures and the maturation of relevant neural circuits, rather than treating DAT as an isolated biological determinant.

The hypothesis further predicts that treatment responsiveness may vary according to developmental stage because the neural circuits contributing to ADHD symptom expression continue to mature across childhood, adolescence, and adulthood. Consequently, prospective longitudinal studies are needed to determine whether pharmacological and non-pharmacological interventions differ in their effectiveness across developmental periods and whether any observed differences are associated with developmental changes in dopaminergic function or neural circuit maturation (68, 83, 84). Such studies should include stimulant and non-stimulant treatments, including methylphenidate, amphetamine-based medications, atomoxetine, guanfacine, and clonidine, to determine whether developmental differences in treatment response vary according to pharmacological mechanism. Repeated clinical, cognitive, and neurobiological assessments will be important for distinguishing developmental effects from those attributable to symptom severity, treatment exposure, comorbidity, or other potential confounding factors.

Sex specific investigations should likewise be prioritized, as accumulating evidence indicates that females with ADHD may exhibit distinct developmental trajectories, symptom profiles, hormonal influences, and treatment responses despite comparable or greater genetic liability (97–99). Determining how these factors interact with developmental changes in dopaminergic and other neurochemical systems may provide further insight into the biological basis of clinical heterogeneity.

More broadly, the proposed framework underscores the need for large scale, multimodal longitudinal studies that follow individuals from childhood into adulthood. Integrating neuroimaging, electrophysiology, molecular profiling, genetics, behavioral phenotyping, and developmental pharmacology within the same longitudinal cohorts would enable direct evaluation of whether DAT related dysregulation is associated with developmental changes in neural circuitry, symptom expression, and functional outcomes over time. Such study designs would provide a more rigorous test of the proposed hypothesis than cross-sectional investigations by directly examining developmental trajectories within individuals. Importantly, these studies should evaluate DAT related measures alongside noradrenergic, glutamatergic, and GABAergic markers to determine whether developmental changes in DAT are associated with broader neurochemical interactions and whether their functional consequences depend on the maturation state of the relevant neural circuits.

Beyond evaluating the Developmental Dopamine Trajectory Hypothesis, these approaches may facilitate the identification of developmental biomarkers associated with ADHD heterogeneity and improve understanding of how neurobiological changes relate to evolving clinical manifestations across the lifespan (91, 93, 98).

## 3 Conclusion

ADHD is increasingly recognized as a lifespan neurodevelopmental disorder characterized by both symptom persistence and substantial changes in clinical expression across development. The Developmental Dopamine Trajectory Hypothesis proposes that these seemingly divergent features may arise from an interaction between persistent DAT dysregulation and the sequential maturation of frontostriatal, mesolimbic, and mesocortical circuits. Within this framework, developmental timing is a key explanatory variable as the functional consequences of DAT related dopaminergic dysregulation may depend on the maturation state of the neural circuits on which it acts. Thus, age dependent shifts from childhood hyperactivity, impulsivity, and attentional instability to adolescent reward dysregulation and emotional difficulties, and ultimately to adult executive dysfunction, motivational impairment, and internal restlessness may reflect evolving consequences of dopaminergic dysregulation within changing neural contexts rather than the emergence of distinct disorders.

By conceptualizing ADHD through the Developmental Dopamine Trajectory Hypothesis rather than as a static dopaminergic deficit, the framework provides a parsimonious model for integrating symptom heterogeneity, fluctuating developmental trajectories, and potential age dependent differences in treatment response. Its principal contribution is therefore not simply to propose another dopamine centered account of ADHD, but to place developmental timing and neural circuit maturation at the center of how persistent DAT related vulnerability may be translated into changing clinical phenotypes. Drawing together evidence from longitudinal clinical studies, neuroimaging, electrophysiology, pharmacology, genetics, and translational DAT models, it offers a translational bridge between developmental neuroscience and clinical ADHD research.

Although the proposed framework remains to be empirically validated, it generates clear, testable predictions regarding symptom transformation, biomarker evolution, and developmental differences in treatment responsiveness. Future multimodal longitudinal studies, including longitudinal PET or SPECT assessment of DAT availability, will be essential to determine whether developmental changes in DAT related signaling correspond to changes in neural circuit maturation and clinical expression across the lifespan. Such studies should also examine these relationships within the broader context of noradrenergic, glutamatergic, and GABAergic signaling. If supported, this developmental framework could inform the design of longitudinal ADHD studies, the identification of developmentally sensitive biomarkers, and the development of age informed treatment strategies.

## Data Availability

The original contributions presented in the study are included in the article/supplementary material. Further inquiries can be directed to the corresponding author.

## References

[1] Folego-Temoteo I Lima YC Grevet EH Vidor MV de A Tavares ME da Silva BS . A comprehensive overview of the biological foundations of ADHD. Biosci Rep. (2026) 46:BSR20254061. doi: 10.1042/bsr20254061 42267530 PMC13259830

[2] Cortese S Bellgrove MA Brikell I Franke B Goodman DW Hartman CA . Attention‐deficit/hyperactivity disorder (ADHD) in adults: evidence base, uncertainties and controversies. World Psychiatry. (2025) 24:347–71. doi: 10.1002/wps.21374 40948064 PMC12434367

[3] Henning CT Summerfeldt LJ Parker JD . Longitudinal associations between symptoms of ADHD and life success: From emerging adulthood to early middle adulthood. J Attention Disord. (2024) 28:1139–51. doi: 10.1177/10870547241239148 38504446 PMC11016205

[4] Skogli EW Orm S Fossum IN Andersen PN Øie MG . Attention-deficit/hyperactivity disorder persistence from childhood into young adult age: a 10-year longitudinal study. Cogn Neuropsychiatry. (2022) 27:447–57. doi: 10.1080/13546805.2022.2123735 36102071

[5] Jiménez-Muñoz L Lopez-Fernandez O Peñuelas-Calvo I Delgado-Gómez D Miguélez-Fernández C López-González S . Persistence of ADHD into adulthood and associated factors: a prospective study. Psiquiatría Biológica. (2025) 32:100529. doi: 10.1016/j.psiq.2024.100529

[6] Van Meter AR Sibley MH Vandana P Birmaher B Fristad MA Horwitz S . The stability and persistence of symptoms in childhood-onset ADHD. Eur Child Adolesc Psychiatry. (2024) 33:1163–70. doi: 10.1007/s00787-023-02235-3 37270740 PMC12490748

[7] McKay CC De Jesus AV Peterson O Leibenluft E Kircanski K . Cross-sectional and longitudinal relations among irritability, attention-deficit/hyperactivity disorder symptoms, and inhibitory control. J Am Acad Child Adolesc Psychiatry. (2024) 63:1014–23. doi: 10.1016/j.jaac.2023.10.015 38272350 PMC13348862

[8] Chaturvedi S . Impact of diagnostic uncertainty in modern psychiatry. Physician. (2025) 9:1. doi: 10.38192/1.9.3.3

[9] Ging-Jehli NR Pine DS . From symptom-based heterogeneity to mechanism-based profiling in youth ADHD: the promise of computational psychiatry. Neuropsychopharmacology. (2026) 51:234–43. doi: 10.1038/s41386-025-02254-5 41028231 PMC12618515

[10] Szymaniak K Bell E Shivakumar G Malhi GS . Breaking down the ADHD construct to build a valid diagnosis. Acta Neuropsychiatr. (2026) 38(e44):1–15. doi: 10.1017/neu.2026.10086 42168168 PMC13280515

[11] Norman LJ Sudre G Bouyssi-Kobar M Jiao M Gligorovic S Jean J . Cross-sectional mega-analysis of resting-state alterations associated with autism and attention-deficit/hyperactivity disorder in children and adolescents. Nat Ment Health. (2025) 3:709–23. doi: 10.1038/s44220-025-00431-5 41446723 PMC12724538

[12] Kofler MJ Soto EF Singh LJ Harmon SL Jaisle E Smith JN . Executive function deficits in attention-deficit/hyperactivity disorder and autism spectrum disorder. Nat Rev Psychol. (2024) 3:701–19. doi: 10.1038/s44159-024-00350-9 39429646 PMC11485171

[13] Sonuga-Barke EJ . The dual pathway model of AD/HD: an elaboration of neuro-developmental characteristics. Neurosci Biobehav Rev. (2003) 27:593–604. doi: 10.1016/j.neubiorev.2003.08.005 14624804

[14] Sergeant J . The cognitive-energetic model: an empirical approach to attention-deficit hyperactivity disorder. Neurosci Biobehav Rev. (2000) 24:7–12. doi: 10.1016/s0149-7634(99)00060-3 10654654

[15] Hinshaw SP . Attention deficit hyperactivity disorder (ADHD): controversy, developmental mechanisms, and multiple levels of analysis. Annu Rev Clin Psychol. (2018) 14:291–316. doi: 10.1146/annurev-clinpsy-050817-084917 29220204

[16] Palmini A . Attention-deficit/hyperactivity disorder (ADHD) in adults: a multilayered approach to a serious disorder of inattention to the future. Arquivos Neuro-Psiquiatria. (2024) 82:001–12. doi: 10.1055/s-0044-1791513 39357854 PMC11500298

[17] MacDonald HJ Kleppe R Szigetvari PD Haavik J . The dopamine hypothesis for ADHD: An evaluation of evidence accumulated from human studies and animal models. Front Psychiatry. (2024) 15:1492126. doi: 10.3389/fpsyt.2024.1492126 39619336 PMC11604610

[18] Custodio RJP Hengstler JG Cheong JH Kim HJ Wascher E Getzmann S . Adult ADHD: it is old and new at the same time–what is it? Rev Neurosci. (2024) 35:225–41. doi: 10.1515/revneuro-2023-0071 37813870

[19] Moffitt TE Houts R Asherson P Belsky DW Corcoran DL Hammerle M . Is adult ADHD a childhood-onset neurodevelopmental disorder? Evidence from a four-decade longitudinal cohort study. Am J Psychiatry. (2015) 172:967–77. doi: 10.1176/appi.ajp.2015.14101266 25998281 PMC4591104

[20] El Archi S Barrault S Garcia M Branger S Maugé D Ballon N . Adult ADHD diagnosis, symptoms of impulsivity, and emotional dysregulation in a clinical sample of outpatients consulting for a behavioral addiction. J Attention Disord. (2023) 27:731–42. doi: 10.1177/10870547231161336 36945199

[21] Martz E Weiner L Weibel S . Identifying different patterns of emotion dysregulation in adult ADHD. Borderline Pers Disord Emotion Dysregulation. (2023) 10:28. doi: 10.1186/s40479-023-00235-y 37743484 PMC10519076

[22] Sherman AL Stamates AL Weyandt L . The association between internal restlessness, drinking motives, and alcohol use outcomes in college students. J Attention Disord. (2025) 29:674–83. doi: 10.1177/10870547251330038 40145566 PMC12151747

[23] de Melo IH Franca G . High functioning, yet high suffering-the need to incorporate invisible struggles in adult ADHD diagnostic assessment/criteria. Front Psychiatry. (2026) 17:1813029. doi: 10.1111/j.1749-6632.2001.tb05777.x 42232996 PMC13224461

[24] Shaw P Stringaris A Nigg J Leibenluft E . Emotion dysregulation in attention deficit hyperactivity disorder. Am J Psychiatry. (2014) 171:276–93. doi: 10.1176/appi.focus.140102 24480998 PMC4282137

[25] Isaac V Lopez V Escobar MJ . Arousal dysregulation and executive dysfunction in attention deficit hyperactivity disorder (ADHD). Front Psychiatry. (2024) 14:1336040. doi: 10.3389/fpsyt.2023.1336040 38298926 PMC10827919

[26] Biederman J DiSalvo ML Hutt Vater CR Woodworth KY Faraone SV . Toward operationalizing executive function deficits in adults with ADHD using the Behavior Rating Inventory of Executive Function—Adult Version (BRIEF-A). J Clin Psychiatry. (2023) 84:22m14530. doi: 10.4088/jcp.22m14530 36416743

[27] Bodalski EA Flory K Meinzer MC . A scoping review of factors associated with emotional dysregulation in adults with ADHD. J Attention Disord. (2023) 27:1540–58. doi: 10.1177/10870547231187148 37470198

[28] Soler-Gutiérrez A-M Pérez-González J-C Mayas J . Evidence of emotion dysregulation as a core symptom of adult ADHD: A systematic review. PloS One. (2023) 18:e0280131. doi: 10.1371/journal.pone.0280131 36608036 PMC9821724

[29] Chua IJJ Salmon C Vinnicombe J Bowen J McNicholas F Adamis D . ADHD symptom manifestation in adulthood: moving beyond conceptualisations of inattention and hyperactivity/impulsivity. Irish J psychol Med. (2026), 1–8. doi: 10.1017/ipm.2026.10175 41640011

[30] Koirala S Grimsrud G Mooney MA Larsen B Feczko E Elison JT . Neurobiology of attention-deficit hyperactivity disorder: historical challenges and emerging frontiers. Nat Rev Neurosci. (2024) 25:759–75. doi: 10.1038/s41583-024-00869-z 39448818 PMC13404203

[31] Klein MO Battagello DS Cardoso AR Hauser DN Bittencourt JC Correa RG . Dopamine: functions, signaling, and association with neurological diseases. Cell Mol Neurobiol. (2019) 39:31–59. doi: 10.1007/s10571-018-0632-3 30446950 PMC11469830

[32] Tripp G Wickens J . Using rodent data to elucidate dopaminergic mechanisms of ADHD: Implications for human personality. Pers Neurosci. (2024) 7:e2. doi: 10.1017/pen.2023.12 38384667 PMC10877278

[33] Shastri GG Sudre G Ahn K Jung B Kolachana B Auluck PK . Cortico-striatal differences in the epigenome in attention-deficit/hyperactivity disorder. Transl Psychiatry. (2024) 14:189. doi: 10.1038/s41398-024-02896-x 38605038 PMC11009227

[34] de la Peña JB Dela Peña IJ Custodio RJ Botanas CJ Kim HJ Cheong JH . Exploring the validity of proposed transgenic animal models of attention-deficit hyperactivity disorder (ADHD). Mol Neurobiol. (2018) 55:3739–54. doi: 10.1007/s12035-017-0608-1 28534274

[35] Meneses A Perez-Garcia G Ponce-Lopez T Tellez R Gallegos-Cari A Castillo C . Spontaneously hypertensive rat (SHR) as an animal model for ADHD: a short overview. Rev Neurosci. (2011) 22(3):365–71. doi: 10.1515/rns.2011.024 21591908

[36] Sagvolden T Johansen EB Wøien G Walaas SI Storm-Mathisen J Bergersen LH . The spontaneously hypertensive rat model of ADHD–the importance of selecting the appropriate reference strain. Neuropharmacology. (2009) 57:619–26. doi: 10.1016/j.neuropharm.2009.08.004 19698722 PMC2783904

[37] Efimova EV Gainetdinov RR Budygin EA Sotnikova TD . Dopamine transporter mutant animals: a translational perspective. J Neurogenet. (2016) 30:5–15. doi: 10.3109/01677063.2016.1144751 27276191 PMC4902158

[38] Savchenko A Targa G Fesenko Z Leo D Gainetdinov RR Sukhanov I . Dopamine transporter deficient rodents: perspectives and limitations for neuroscience. Biomolecules. (2023) 13:806. doi: 10.3390/biom13050806 37238676 PMC10216310

[39] Dluzen DE Ji J McDermott JL . Age-related changes in nigrostriatal dopaminergic function in heterozygous mutant dopamine transporter knock-out mice. Neurosci Lett. (2010) 476:66–9. doi: 10.1016/j.neulet.2010.04.004 20382201

[40] Ji J Dluzen DE . Sex differences in striatal dopaminergic function within heterozygous mutant dopamine transporter knock-out mice. J Neural Transm. (2008) 115:809–17. doi: 10.1007/s00702-007-0017-0 18197357

[41] Mazei-Robison MS Bowton E Holy M Schmudermaier M Freissmuth M Sitte HH . Anomalous dopamine release associated with a human dopamine transporter coding variant. J Neurosci. (2008) 28:7040–6. doi: 10.1523/jneurosci.0473-08.2008 18614672 PMC2573963

[42] Mergy MA Gowrishankar R Gresch PJ Gantz SC Williams J Davis GL . The rare DAT coding variant Val559 perturbs DA neuron function, changes behavior, and alters *in vivo* responses to psychostimulants. Proc Natl Acad Sci. (2014) 111:E4779–88. doi: 10.1073/pnas.1417294111 25331903 PMC4226116

[43] Davis GL Stewart A Stanwood GD Gowrishankar R Hahn MK Blakely RD . Functional coding variation in the presynaptic dopamine transporter associated with neuropsychiatric disorders drives enhanced motivation and context-dependent impulsivity in mice. Behav Brain Res. (2018) 337:61–9. doi: 10.1016/j.bbr.2017.09.043 28964912 PMC5645257

[44] Gowrishankar R Gresch PJ Davis GL Katamish RM Riele JR Stewart AM . Region-specific regulation of presynaptic dopamine homeostasis by D2 autoreceptors shapes the *in vivo* impact of the neuropsychiatric disease-associated DAT variant Val559. J Neurosci. (2018) 38:5302–12. doi: 10.1523/jneurosci.0055-18.2018 29739866 PMC5990980

[45] Stewart A Davis GL Areal LB Rabil MJ Tran V Mayer FP . Male DAT Val559 mice exhibit compulsive behavior under devalued reward conditions accompanied by cellular and pharmacological changes. Cells. (2022) 11:4059. doi: 10.3390/cells11244059 36552823 PMC9777203

[46] Sharma R Kour A Dewangan HK . Enhancements in parkinson's disease management: leveraging levodopa optimization and surgical breakthroughs. Curr Drug Targets. (2025) 26:17–32. doi: 10.2174/0113894501319817240919103802 39350551

[47] Cherkasova M Sulla EM Dalena KL Pondé MP Hechtman L . Developmental course of attention deficit hyperactivity disorder and its predictors. J Can Acad Child Adolesc Psychiatry. (2013) 22:47. PMC3565715 23390433

[48] Martínez L Prada E Satler C Tavares MCH Tomaz C . Executive dysfunctions: the role in attention deficit hyperactivity and post-traumatic stress neuropsychiatric disorders. Front Psychol. (2016) 7:206398. doi: 10.3389/fpsyg.2016.01230 PMC4993788 27602003

[49] Thapar A Cooper M Jefferies R Stergiakouli E . What causes attention deficit hyperactivity disorder? Arch Dis Childhood. (2012) 97:260–5. doi: 10.1136/archdischild-2011-300482 21903599 PMC3927422

[50] Volkow ND Wang GJ Kollins SH Wigal TL Newcorn JH Telang F . Evaluating dopamine reward pathway in ADHD: clinical implications. Jama. (2009) 302:1084–91. doi: 10.1001/jama.2009.1308 19738093 PMC2958516

[51] Volkow ND Wang GJ Newcorn JH Kollins SH Wigal TL Telang F . Motivation deficit in ADHD is associated with dysfunction of the dopamine reward pathway. Mol Psychiatry. (2011) 16:1147–54. doi: 10.1038/mp.2010.97 20856250 PMC3010326

[52] Shaw P . Attention-deficit/hyperactivity disorder and the battle for control of attention. J Am Acad Child Adolesc Psychiatry. (2012) 51:1116–8. doi: 10.1016/j.jaac.2012.06.016 23101738

[53] Petrovic P Castellanos FX . Top-down dysregulation—from ADHD to emotional instability. Front Behav Neurosci. (2016) 10:70. doi: 10.3389/fnbeh.2016.00070 27242456 PMC4876334

[54] van Stralen J . Emotional dysregulation in children with attention-deficit/hyperactivity disorder. ADHD Attention Deficit Hyperactivity Disord. (2016) 8:175–87. doi: 10.1007/s12402-016-0199-0 27299358 PMC5110580

[55] Seymour KE Chronis-Tuscano A Halldorsdottir T Stupica B Owens K Sacks T . Emotion regulation mediates the relationship between ADHD and depressive symptoms in youth. J Abnormal Child Psychol. (2012) 40:595–606. doi: 10.1007/s10802-011-9593-4 22113705

[56] Franke B Michelini G Asherson P Banaschewski T Bilbow A Buitelaar JK . Live fast, die young? A review on the developmental trajectories of ADHD across the lifespan. Eur Neuropsychopharmacol. (2018) 28:1059–88. doi: 10.1016/j.euroneuro.2018.08.001 30195575 PMC6379245

[57] Sibley MH Kennedy TM Swanson JM Arnold LE Jensen PS Hechtman LT . Characteristics and predictors of fluctuating attention-deficit/hyperactivity disorder in the multimodal treatment of ADHD (MTA) study. J Clin Psychiatry. (2024) 85:24m15395. doi: 10.4088/jcp.24m15395 39431909

[58] Adamou M Kehagias A Antoniou G . A mathematical framework for modelling the dynamic nature of ADHD symptoms. Front Psychiatry. (2025) 16:1671764. doi: 10.3389/fpsyt.2025.1671764 41584753 PMC12827614

[59] Sibley MH Swanson JM Arnold LE Hechtman LT Owens EB Stehli A . Defining ADHD symptom persistence in adulthood: optimizing sensitivity and specificity. J Child Psychol Psychiatry. (2017) 58:655–62. doi: 10.1111/jcpp.12620 27642116 PMC5809153

[60] Graziano PA Garcia A . Attention-deficit hyperactivity disorder and children's emotion dysregulation: A meta-analysis. Clin Psychol Rev. (2016) 46:106–23. doi: 10.1016/j.cpr.2016.04.011 27180913

[61] Viering T Hoekstra PJ Philipsen A Naaijen J Dietrich A Hartman CA . Emotion dysregulation and integration of emotion-related brain networks affect intraindividual change in ADHD severity throughout late adolescence. NeuroImage. (2021) 245:118729. doi: 10.1016/j.neuroimage.2021.118729 34813971

[62] del Campo N Fryer TD Hong YT Smith R Brichard L Acosta-Cabronero J . A positron emission tomography study of nigro-striatal dopaminergic mechanisms underlying attention: implications for ADHD and its treatment. Brain. (2013) 136:3252–70. doi: 10.1093/brain/awt263 24163364 PMC4125626

[63] Ijomone OK Oria RS Ijomone OM Aschner M Bornhorst J . Dopaminergic perturbation in the aetiology of neurodevelopmental disorders. Mol Neurobiol. (2025) 62:2420–34. doi: 10.1007/s12035-024-04418-8 39110391 PMC11772124

[64] Gerez-Malo M Tello A Martin-Salas MJ Castanedo L Mendizábal A Meneses Luna O . Neurophysiological findings in Attention Deficit Hyperactivity Disorder, a Pandora’s box with therapeutic implications. Explor Neuroprotective Ther. (2025) 5:100499. doi: 10.37349/ent.2025.100499

[65] Albrecht B Uebel-von Sandersleben H Gevensleben H Rothenberger A . Pathophysiology of ADHD and associated problems—starting points for NF interventions? Front Hum Neurosci. (2015) 9:2015. doi: 10.3389/fnhum.2015.00359 26157377 PMC4478393

[66] Véronneau-Veilleux F Robaey P Ursino M Nekka F . A mechanistic model of ADHD as resulting from dopamine phasic/tonic imbalance during reinforcement learning. Front Comput Neurosci. (2022) 16:849323. doi: 10.3389/fncom.2022.849323 35923915 PMC9342605

[67] Cortese S Adamo N Del Giovane C Mohr-Jensen C Hayes AJ Carucci S . Comparative efficacy and tolerability of medications for attention-deficit hyperactivity disorder in children, adolescents, and adults: a systematic review and network meta-analysis. Lancet Psychiatry. (2018) 5:727–38. doi: 10.1016/s2215-0366(18)30269-4 30097390 PMC6109107

[68] Chang J-C Lin H-Y Gau S-F . Age-dependent effects of cumulative methylphenidate exposure on brain structure and symptom amelioration in youth with ADHD: A longitudinal MRI study. Prog Neuro-Psychopharmacol Biol Psychiatry. (2025) 140:111429. doi: 10.1016/j.pnpbp.2025.111429 40527386

[69] Blader JC Pliszka SR Jensen PS Schooler NR Kafantaris V . Stimulant-responsive and stimulant-refractory aggressive behavior among children with ADHD. Pediatrics. (2010) 126:e796–806. doi: 10.1542/peds.2010-0086 20837589 PMC2956067

[70] Bluschke A Zink N Mückschel M Roessner V Beste C . A novel approach to intra-individual performance variability in ADHD. Eur Child Adolesc Psychiatry. (2021) 30:733–45. doi: 10.1007/s00787-020-01555-y 32410131 PMC8060200

[71] Kratochvil CJ Vaughan BS Harrington MJ Burke WJ . Atomoxetine: a selective noradrenaline reuptake inhibitor for the treatment of attention-deficit/hyperactivity disorder. Expert Opin Pharmacother. (2003) 4:1165–74. doi: 10.1517/eoph.4.7.1165.21068 12831341

[72] Neuchat EE Bocklud BE Kingsley K Barham WT Luther PM Ahmadzadeh S . The role of alpha-2 agonists for attention deficit hyperactivity disorder in children: A review. Neurol Int. (2023) 15:697–707. doi: 10.3390/neurolint15020043 37218982 PMC10204383

[73] Noah AA Sedky HE . New frontiers in pharmacological treatment of attention-deficit hyperactivity disorder. Naunyn-Schmiedeberg's Arch Pharmacol. (2025) 398:15025–35. doi: 10.1007/s00210-025-04328-z 40478337 PMC12552245

[74] Capuzzi E Caldiroli A Auxilia AM Borgonovo R Capellazzi M Clerici M . Biological predictors of treatment response in adult attention deficit hyperactivity disorder (ADHD): A systematic review. J Personalized Med. (2022) 12:1742. doi: 10.3390/jpm12101742 36294881 PMC9605680

[75] Liman C Schein J Wu A Huang X Thadani S Childress A . Real world analysis of treatment change and response in adults with attention-deficit/hyperactivity disorder (ADHD) alone and with concomitant psychiatric comorbidities: results from an electronic health record database study in the United States. BMC Psychiatry. (2024) 24:618. doi: 10.1186/s12888-024-05994-8 39285361 PMC11406735

[76] Tripodi B Carbone MG Matarese I Rizzato R Della Rocca F De Dominicis F . Effectiveness of pharmacological treatments for adult ADHD on psychiatric comorbidity: A systematic review. J Clin Med. (2025) 14:8848. doi: 10.3390/jcm14248848 41464755 PMC12734097

[77] Faraone SV Bellgrove MA Brikell I Cortese S Hartman CA Hollis C . Attention-deficit/hyperactivity disorder. Nat Rev Dis Primers. (2024) 10:11. doi: 10.1016/b978-0-12-801238-3.05581-1 38388701

[78] Norman LJ Price J Ahn K Sudre G Sharp W Shaw P . Longitudinal trajectories of childhood and adolescent attention deficit hyperactivity disorder diagnoses in three cohorts. eClinicalMedicine. (2023) 60:102021. doi: 10.1016/j.eclinm.2023.102021 37333663 PMC10272308

[79] Williamson P Koro-Ljungberg ME Bussing R . Analysis of critical incidents and shifting perspectives: transitions in illness careers among adolescents with ADHD. Qual Health Res. (2009) 19:352–65. doi: 10.1177/1049732308329683 19224878 PMC3623297

[80] Hurjui IA Hurjui RM Hurjui LL Serban IL Dobrin I Apostu M . Biomarkers and neuropsychological tools in attention-deficit/hyperactivity disorder: from subjectivity to precision diagnosis. Medicina. (2025) 61:1211. doi: 10.3390/medicina61071211 40731840 PMC12300379

[81] Michelini G Norman LJ Shaw P Loo SK . Treatment biomarkers for ADHD: Taking stock and moving forward. Transl Psychiatry. (2022) 12:444. doi: 10.1038/s41398-022-02207-2 36224169 PMC9556670

[82] Cha KS Kim B Lee JY Yoo H . Prediction of attention deficit hyperactivity disorder using the comprehensive attention test: a large-scale machine learning approach. Front Psychiatry. (2025) 16:2025. doi: 10.3389/fpsyt.2025.1574615 40496821 PMC12149198

[83] Schrantee A Tamminga HGT Bouziane C Bottelier MA Bron EE Mutsaerts HJMM . Age-dependent effects of methylphenidate on the human dopaminergic system in young vs adult patients with attention-deficit/hyperactivity disorder: A randomized clinical trial. JAMA Psychiatry. (2016) 73:955–62. doi: 10.1001/jamapsychiatry.2016.1572 27487479 PMC5267166

[84] Solleveld MM Schrantee A Puts NAJ Reneman L Lucassen PJ . Age-dependent, lasting effects of methylphenidate on the GABAergic system of ADHD patients. NeuroImage: Clin. (2017) 15:812–8. doi: 10.1016/j.nicl.2017.06.003 28725548 PMC5506880

[85] Friedel E Vijayakumar N Staniland L Silk TJ . Puberty and ADHD: A scoping review and framework for future research. Clin Psychol Rev. (2025) 117:102567. doi: 10.1016/j.cpr.2025.102567 40058298

[86] Larsen B Luna B . Adolescence as a neurobiological critical period for the development of higher-order cognition. Neurosci Biobehav Rev. (2018) 94:179–95. doi: 10.1016/j.neubiorev.2018.09.005 30201220 PMC6526538

[87] Biezonski D Shah R Krivko A Cha J Guilfoyle DN Hrabe J . Longitudinal magnetic resonance imaging reveals striatal hypertrophy in a rat model of long-term stimulant treatment. Transl Psychiatry. (2016) 6:e884–4. doi: 10.1038/tp.2016.158 27598968 PMC5048200

[88] Corona JC . Rodent research of attention-deficit/hyperactivity disorder: insights into widely used animal models. Lab Anim Res. (2025) 41:24. doi: 10.1186/s42826-025-00255-5 40988072 PMC12455772

[89] Krause J . SPECT and PET of the dopamine transporter in attention-deficit/hyperactivity disorder. Expert Rev Neurother. (2008) 8:611–25. doi: 10.1586/14737175.8.4.611 18416663

[90] Chen IC Chang CL Huang IW Chang MH Ko LW . Electrophysiological functional connectivity and complexity reflecting cognitive processing speed heterogeneity in young children with ADHD. Psychiatry Res. (2024) 340:116100. doi: 10.1016/j.psychres.2024.116100 39121760

[91] Gao Z Duberg K Warren SL Zheng L Hinshaw SP Menon V . Reduced temporal and spatial stability of neural activity patterns predict cognitive control deficits in children with ADHD. Nat Commun. (2025) 16:2346. doi: 10.1038/s41467-025-57685-x 40057478 PMC11890578

[92] Khachidze I Parkosadze K Zhamureli N Khachidze A Advadze M . Electrophysiological evaluation of cognitive intervention outcomes in children with attention deficit/hyperactivity disorder. Front Neurosci. (2026) 20:2026. doi: 10.3389/fnins.2026.1777670 41987928 PMC13076253

[93] Karabulut OB Unal D . Proteomic findings in ADHD: A systematic review. Int J Dev Neurosci. (2025) 85:e70057. doi: 10.1002/jdn.70057 41236087

[94] Liu J . Proteome-wide association studies have predicted that the protein abundance of LSM6, GMPPB, ICA1L, and CISD2 is associated with attention-deficit/hyperactivity disorder. Eur Child Adolesc Psychiatry. (2025) 34:721–8. doi: 10.1007/s00787-024-02517-4 38954053

[95] Custodio RJP Sayson LV Cho A Jung H Ortiz DM Lee HJ . Unraveling predominantly inattentive ADHD (ADHD-PI): insights from proteomic analysis of the striatum of thyroid hormone-responsive protein (THRSP)–overexpressing mice. Mol Neurobiol. (2025) 62:13225–49. doi: 10.1007/s12035-025-05031-z 40493342 PMC12433356

[96] Custodio RJP Kim HJ Kim J Ortiz DM Kim M Buctot D . Hippocampal dentate gyri proteomics reveals Wnt signaling involvement in the behavioral impairment in the THRSP-overexpressing ADHD mouse model. Commun Biol. (2023) 6:55. doi: 10.1038/s42003-022-04387-5 36646879 PMC9842619

[97] Guagliardo S Lundberg M Schork A Cox N Shuey M . Evaluating the impact of biological sex on ADHD presentation, prevalence, and genetic risk. Eur Neuropsychopharmacol. (2024) 87:30. doi: 10.1016/j.euroneuro.2024.08.07642574925

[98] Kooij JJS . New developments and potential future research directions in adult ADHD. World Psychiatry. (2025) 24:381–2. doi: 10.65031/kcvv5502 PMC12434362 40948075

[99] Martin J Taylor MJ Rydell M Riglin L Eyre O Lu Y . Sex-specific manifestation of genetic risk for attention deficit hyperactivity disorder in the general population. J Child Psychol Psychiatry. (2018) 59:908–16. doi: 10.1007/978-1-4419-1428-6_268 PMC6055636 29451303

